# Single-incision trans-abdominal preperitoneal mesh hernioplasty

**DOI:** 10.4103/0972-9941.72376

**Published:** 2011

**Authors:** Prabal Roy, Anushtup De

**Affiliations:** Department of General and Minimally Invasive Surgery, Asian Institute of Medical Sciences, Faridabad, India

**Keywords:** Single-incision mesh hernioplasty, single-incision laparoscopic surgery, transabdominal preperitoneal

## Abstract

Single-incision laparoscopy is being used to carry out a wide variety of laparoscopic operations since its introduction in 2007. Various case reports and studies have demonstrated the safety and feasibility of single-incision laparoscopic transabdominal preperitoneal (TAPP) and totally extra-peritoneal mesh hernioplasty. However, till date, its apparent advantages have been mainly cosmetic and related to patient satisfaction. We have been performing single-incision laparoscopic TAPP mesh hernioplasty since June 2009 using conventional laparoscopic instruments. Here, we describe our technique that is aimed at standardising the method.

## INTRODUCTION

Advances in laparoscopic technique have ushered in an opportunity to continuously improve and improvise upon many surgical procedures. Over the past few years, there has been a trend toward further minimising minimally invasive approaches with the introduction of single-incision and natural orifice laparoscopic surgery. Navarra *et al*.[1] had initially reported on the technical feasibility of laparoscopic cholecystectomy through a single, transumbilical incision. This was followed by the first few cases of cholecystectomy using the transvaginal and transcolonic routes.[2,3] However, some of the disadvantages of Natural Orifice Transluminal Endoscopic Surgery (NOTES) have not allowed this technique to be widely practiced. These include the hesitation on the part of the surgeon in transgressing a sensitive mucosal barrier, expensive instrumentation and a steep learning curve associated with the technical challenge of a new approach. It is being performed in only a few centres worldwide. While NOTES is still struggling to find its rightful place, single-incision laparoscopy has generated tremendous interest in the surgical fraternity worldwide, as is evident from a slew of reports being published in the literature since 2008. Rao *et al*.[4] reported a series of single-incision laparoscopic cholecystectomy. This was followed by case reports of single-incision laparoscopic hernia repair – both totally extra-peritoneal and transabdominal preperitoneal (TAPP) procedures.[5,6] In this paper, we describe our technique that is aimed at standardising the method.

### Patient selection and preoperative preparation

All patients diagnosed to have inguinal hernia on clinical examination and who were fit for general anaesthesia were offered to undergo single-incision laparoscopic TAPP mesh hernioplasty. Only the patients with irreducible or complicated hernia were excluded from the study.

## SURGICAL TECHNIQUE

Attempts on single-incision TAPP were made after we had gained significant experience in single-incision laparoscopic cholecystectomy.[7] During this experience, we found that the use of conventional instruments to carry out laparoscopic surgery by single-incision made the procedure easier to adopt. The basic operative steps of single-incision TAPP mesh hernioplasty are similar to that of conventional laparoscopic TAPP mesh hernioplasty.

The patient is placed supine with both hands restrained by his sides. The table is given a 20° Trendelenberg tilt and the side on which the surgery is to be performed is tilted up by about 20°. This helps in reducing the contents of the sac and displaces the intraabdominal viscera away from the operating site. The first assistant handling the camera stands beside the patient’s opposite shoulder and the surgeon stands just caudad.

**Figure 1:**
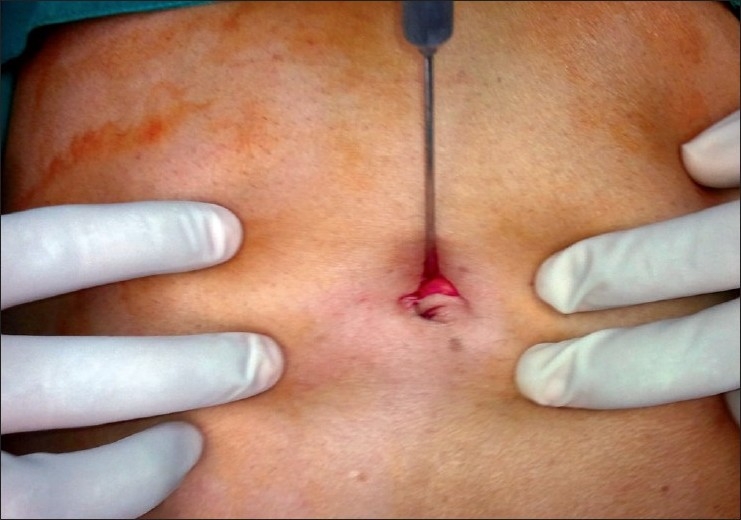
Curvilinear supraumbilical incision

**Figure 2:**
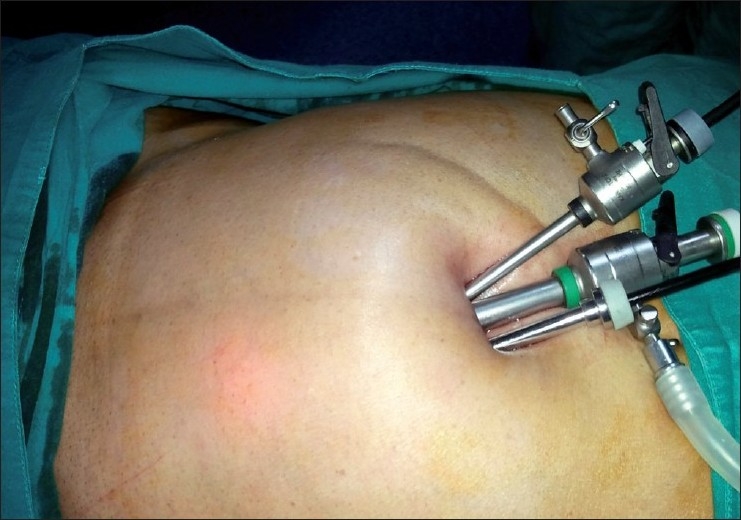
All ports placed through the umbilical incision

**Figure 3:**
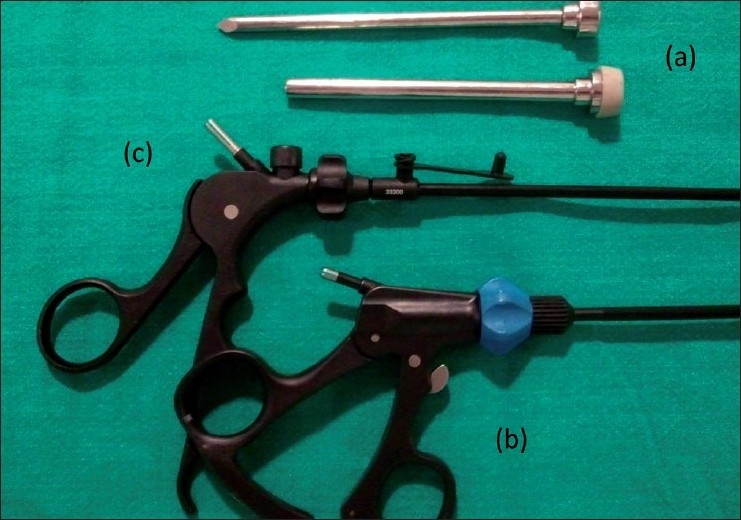
(a) Low-profile valveless port with trocar, (b) instrument with inline cautery connection at the handle compared with other instrument (c)

We make a curvilinear incision of about 20 mm at the cephalic margin of the umbilical crease [Figure 1]. Pneumoperitoneum is established using a Veress needle. We then place a 10-mm port in the midline through which a 45-cm 50° laparoscope (Panoview, Richard Wolf Medical Instruments Corporation, Vernon Hills, IL, USA) is introduced. The peritoneal cavity is carefully inspected for any pathology that might demand conversion to conventional laparoscopy. Two 5-mm ports are then inserted through the same incision by piercing the sheath about 1-cm caudal and lateral on either side of the 10-mm port [Figure 2]. One of the two 5-mm ports is a low-profile valveless port [Figure 3]. This decreases the collision of ports at the common entry point and also improves the manuverability of instruments inside the abdomen. Laparoscopic dissecting and grasping instruments and scissors are passed through the 5-mm ports and triangulated as comfortably as is possible. The authors prefer to use instruments that have an inline contact position for connecting an electrocautery rather than it being at an angle [Figure 3]. This also reduces the cluttering of instruments during the surgery.

**Figure 4:**
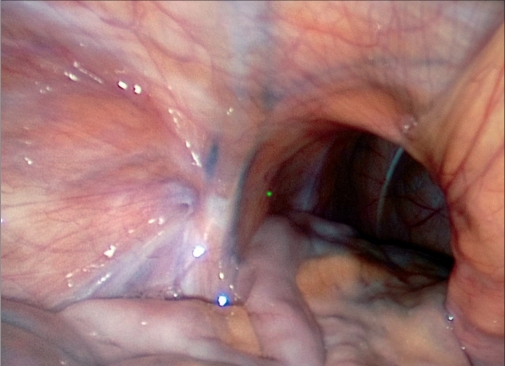
Hernial opening with reduced contents

**Figure 5:**
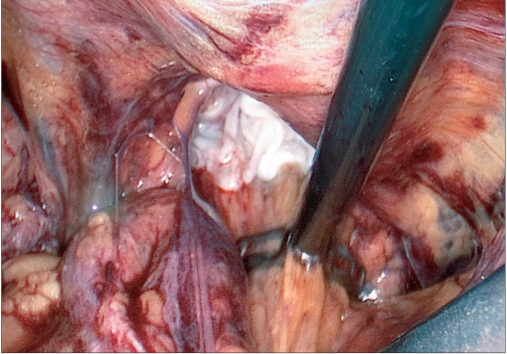
Dissection of hernial sac with separation from pseudosac

**Figure 6:**
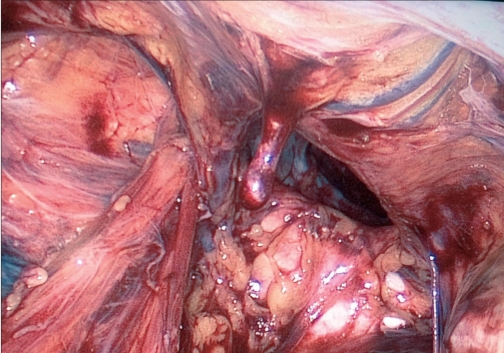
Extent of dissection

**Figure 7:**
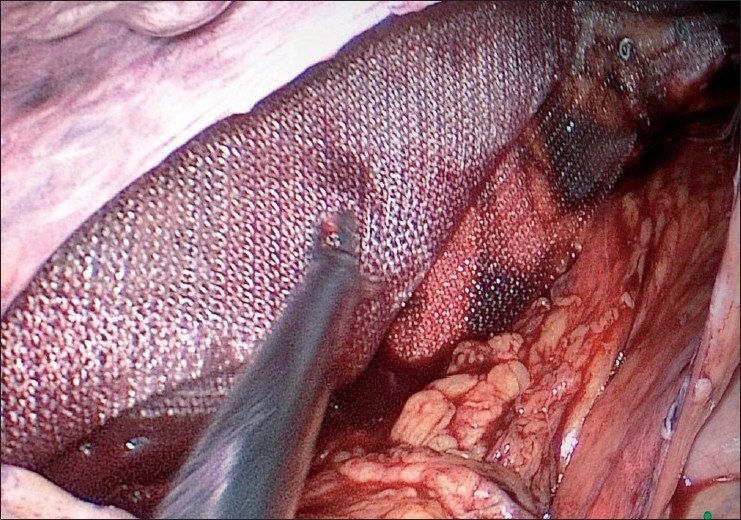
Mesh placed and held with tackers

Contents of the hernial sac are reduced [Figure 4] and then, as with traditional TAPP, using monopolar scissors or an “L”-hook, we create a horizontal peritoneal incision about 2 cm above the hernial defect extending from the medial umbilical ligament to the anterior superior iliac spine. The peritoneum and the sac are meticulously separated from the pseudosac by blunt and sharp dissection, taking care not to allow oozing blood to obscure vision [Figure 5]. Dissection is performed in the avascular cobweb-like areolar tissue. The area of dissection extends till the symphsis pubis in the midline and beyond the anterior superior iliac spine, exposing the psoas muscle in the lateral aspect [Figure 6]. Care is taken to preserve the covering layer of fascia over the psoas muscle in order to protect the nerves running across it. The vas deferens and gonadal vessels are parietalised sufficiently so as to allow placement of a 15 cm × 12 cm mesh [Figure 7]. A note is made of the advantage that the 50-degree 45-cm telescope allows a wider field of vision with simple rotation of its axis.

**Figure 8:**
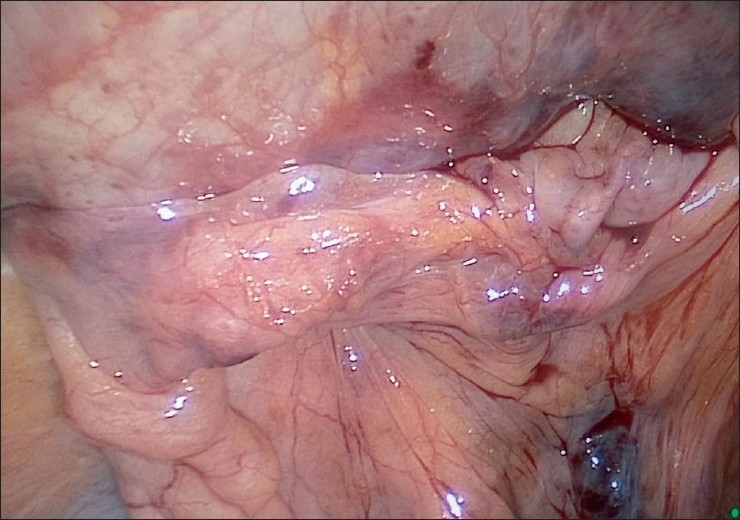
Reperitonization of mesh

The mesh is placed with its long axis in the transverse plane, medially across the pubic symphysis and laterally up to the lateral end of the iliopubic tract. The mesh is fixed to Cooper’s ligament medially and also at the superolateral angle using a tacker device (ProTack, Covidien, Mansfield, OH, USA). The peritoneum is then fixed over the mesh using the tacker device [Figure 8]. Pneumoperitoneum is released under vision. The fascial and skin incisions are closed.

## RESULTS

Forty-two patients underwent single-incision laparoscopic TAPP mesh hernioplasty during the period from June 2009 to March 2010. The average age of the patients was 38 years. Eight of the patients had bilateral hernia and the remaining had unilateral hernia. In two patients, we had laterally sliding hernia on the left side. In five patients, we had partially reducible bowel as content and irreducible omentum was detected in two patients, which could be dissected and reduced safely. The average operative time for unilateral was 49.03 min. The average postoperative duration of stay was 1 day (range, 1–2 days). Injection Diclofenac sodium 75 mg twice daily was given for postoperative analgesia for the duration of stay. None of the patients required additional analgesia. This was followed by Tab Diclofenac twice daily for 2 days. None of the patents in this series needed conversion to conventional laparoscopy. None of the patients reported neuralgia, incisional hernia or any other significant problem in their follow-up (4–13 months).

## CONCLUSION

Rahman *et al*.[5] reported the first case of single-incision TAPP using roticulating instruments. However, they reported loss of tactile feedback compared with nonroticulating instruments. Buschner *et al*.,[8] in their preliminary experience, also reported improved feasibility and safety of single-incision TAPP with a good cosmetic result. Our study corroborates the safety and feasibility of single-incision TAPP mesh hernioplasty. However, the apparent advantage till date appears to be mainly cosmetic and further studies in the form of randomized controlled trials to evaluate its practical benefits and potential risks are necessary.

In the evolution of minimally invasive surgery, single-incision techniques have been introduced in an attempt to reduce invasiveness further. The feasibility of the operation and that it may afford a better cosmesis is no longer in question. Whether it will possibly decrease patient morbidity any further or that the procedure may simply get archived in the history, like the Angelchik’s prosthesis in the treatment of gastroesophageal reflux disease, will become apparent with greater experience. Conversely, just as with experience, standardisation of operations and surmounting the learning curve of conventional laparoscopic surgery, its advantages in comparison with conventional open surgery far outweigh concerns about the increased risk of complications; single-incision laparoscopy may well become the gold standard in times to come.
